# Development and validation of PBPK models for genistein and daidzein for use in a next-generation risk assessment

**DOI:** 10.3389/fphar.2024.1421650

**Published:** 2024-10-03

**Authors:** A. Najjar, D. Lange, C. Géniès, J. Kuehnl, A. Zifle, C. Jacques, E. Fabian, N. Hewitt, A. Schepky

**Affiliations:** ^1^ Beiersdorf AG, Hamburg, Germany; ^2^ Pierre Fabre Dermo-Cosmétique and Personal Care, Toulouse, France; ^3^ Kao Germany GmbH, Darmstadt, Germany; ^4^ BASF SE, Ludwigshafen, Germany; ^5^ Cosmetics Europe, Brussels, Belgium

**Keywords:** daidzein, genistein, PBPK, validation, safety assessment

## Abstract

**Introduction:**

All cosmetic ingredients must be evaluated for their safety to consumers. In the absence of *in vivo* data, systemic concentrations of ingredients can be predicted using Physiologically based Pharmacokinetic (PBPK) models. However, more examples are needed to demonstrate how they can be validated and applied in Next-Generation Risk Assessments (NGRA) of cosmetic ingredients. We used a bottom-up approach to develop human PBPK models for genistein and daidzein for a read-across NGRA, whereby genistein was the source chemical for the target chemical, daidzein.

**Methods:**

An oral rat PBPK model for genistein was built using PK-Sim^®^ and *in vitro* ADME input data. This formed the basis of the daidzein oral rat PBPK model, for which chemical-specific input parameters were used. Rat PBPK models were then converted to human models using human-specific physiological parameters and human *in vitro* ADME data. *In vitro* skin metabolism and penetration data were used to build the dermal module to represent the major route of exposure to cosmetics.

**Results:**

The initial oral rat model for genistein was qualified since it predicted values within 2-fold of measured *in vivo* PK values. This was used to predict plasma concentrations from the *in vivo* NOAEL for genistein to set test concentrations in bioassays. Intrinsic hepatic clearance and unbound fractions in plasma were identified as sensitive parameters impacting the predicted C_max_ values. Sensitivity and uncertainty analyses indicated the developed PBPK models had a moderate level of confidence. An important aspect of the development of the dermal module was the implementation of first-pass metabolism, which was extensive for both chemicals. The final human PBPK model for daidzein was used to convert the *in vitro* PoD of 33 nM (from an estrogen receptor transactivation assay) to an external dose of 0.2% in a body lotion formulation.

**Conclusion:**

PBPK models for genistein and daidzein were developed as a central component of an NGRA read-across case study. This will help to gain regulatory confidence in the use of PBPK models, especially for cosmetic ingredients.

## 1 Introduction

All cosmetic ingredients must be evaluated for their safety to consumers. Assessments involve their potential to cause local effects to the skin or eye and systemic effects (SCCS, 2023). The prediction of systemic effects requires reliable estimations of the distribution and concentration of parent and/or metabolites once they enter the circulation. However, as a result of the full animal testing ban, which came into effect in March 2013, safety assessments of cosmetic ingredients submitted to regulators in the European Union (EU) must now be assessed using non-animal methods (EU, 2009). This in turn places great responsibility on the shoulders of Physiologically Based Pharmacokinetic (PBPK) model developers to build robust models without the need of generating further animal data. These models are used to quantify and predict the absorption, distribution, metabolism and excretion (ADME) properties of chemicals after exposure. For the cosmetics industry, the PBPK models also need to consider dermal exposure since this is the major route of application of cosmetics. Despite the existence of PBPK models for many decades and the use of these in regulatory submissions of pharmaceuticals (Chen et al., 2012; Tan et al., 2020), as well as guidance documents for the characterization and application of these models in risk assessments (OECD, 2021b; OECD, 2021c; WHO. Project H, 2010), more examples are needed to show how they can be validated and applied in Next-Generation Risk Assessments (NGRA) of cosmetic ingredients to gain the confidence of regulatory bodies (Dent et al., 2021).

PBPK models have been key components of the NGRA case studies performed as part of the Cosmetics Europe Long Range Science Strategy (LRSS) Program (Bury et al., 2021; Ouedraogo et al., 2022; OECD, 2021a; Desprez et al., 2018). In these case studies, a Margin of Internal Exposure (MoIE) was derived for the target chemicals, whereby plasma concentrations were estimated using PBPK modelling and compared with a Point of Departure (PoD) derived from *in vitro* bioassays, e.g., the no effect concentrations from transcriptomics assays in cell lines and estrogen, androgen, thyroid and steroidogenic (EATS) assays. In the LRSS case study on phenoxyethanol, a PBPK model was built using a previously published model as the basis but excluded any *in vivo* animal data for further development since it was an *ab initio* cases study, which assumes no *in vivo* data are available (OECD, 2021a). The PBPK model was retrospectively qualified by comparing the predicted values with human clinical data, thus providing a high confidence in the model output. Others have used PBPK models to predict plasma concentrations of cosmetic ingredients after topical application. For example, Baltazar et al. (2020) reported the PBPK model output for coumarin correlated with a high concordance with human clinical data. A PBPK model was developed by Najjar et al. (2021) to support the safety of the UV filter ingredient, homosalate. The initial model was an intravenous (IV) rat PBPK model, which was validated by comparing predicted values with measured values from a legacy rat pharmacokinetics (PK) study [i.e., data from *in vivo* animal studies conducted prior to the full animal testing ban in March 2013 (EU, 2009)]. The IV rat model was subsequently adapted using human *in vitro* data and *in vitro*-to-*in vivo* extrapolation (IVIVE) equations (e.g., converting *in vitro* intrinsic clearance values, expressed as µL/min/million cells, in hepatocytes to predicted *in vivo* hepatic clearance expressed as L/h) to a human whole body PBPK model. Since the most relevant route of exposure of the UV filter was via the skin, a dermal module was developed. As with the coumarin and phenoxyethanol case studies (OECD, 2021a; Baltazar et al., 2020), the performance of the homosalate PBPK model was confirmed by comparing predicted and measured human clinical data.

**FIGURE 1:**
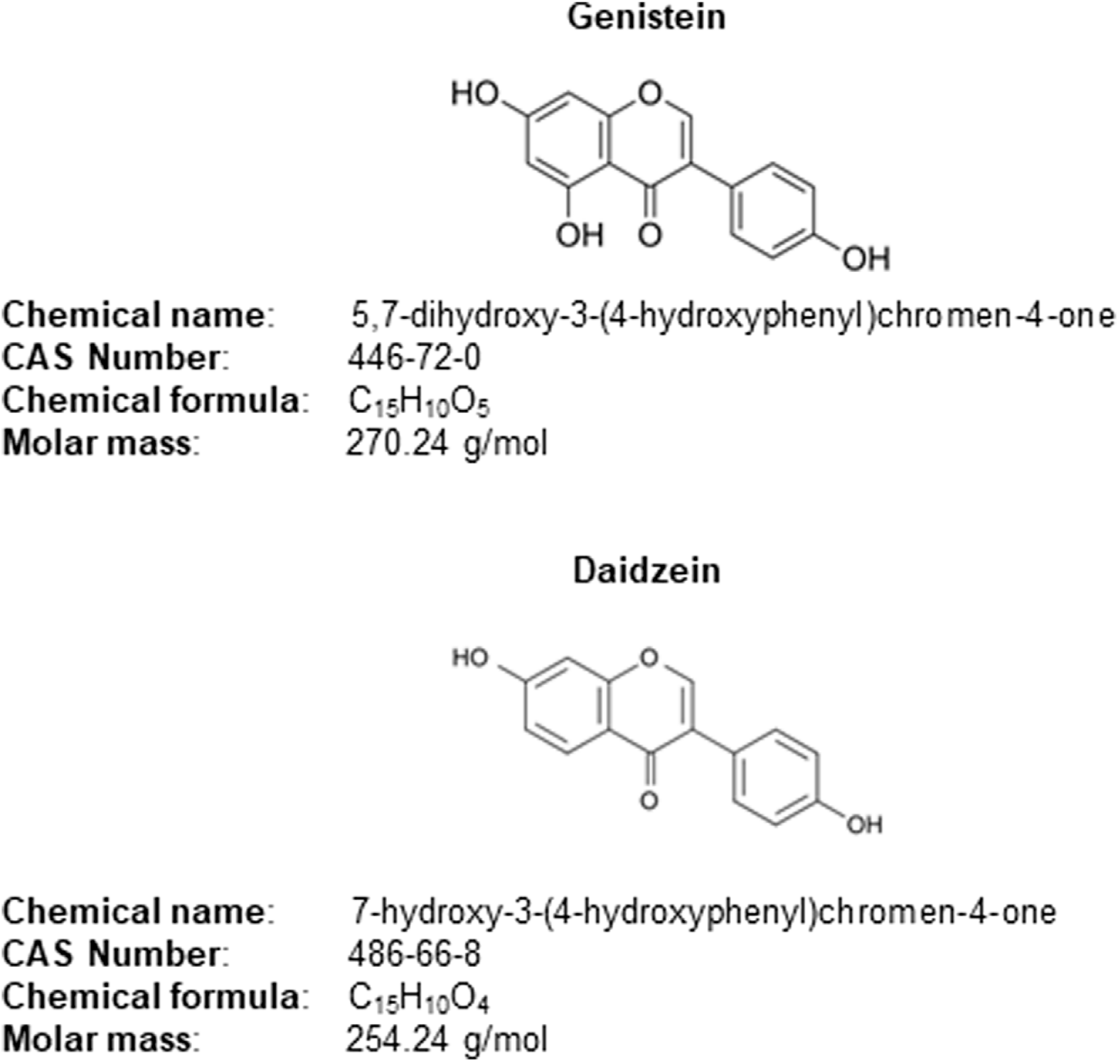
Genistein and daidzein – properties and structure.

Genistein and daidzein are isoflavones and are used as anti-aging components in cosmetic formulations applied to the face and body (SCCS, 2022). These were used in the LRSS study described by Najjar et al. (2024a) to demonstrate how New Approach Methodologies (NAMs), such as *in silico* and *in vitro* models, could be used in a read-across NGRA for cosmetic ingredients, whereby genistein was the source chemical for daidzein (the target chemical) (Najjar et al., 2024a). Genistein has the potential to cause endocrine disruption (SCCS, 2022) and since daidzein has a similar structure (see Figure 1), the latter was evaluated for its potential to also cause endocrine disruption. The bioactivity and safety assessment for both chemicals were based on results from the EATS assay panel, with the most relevant PoDs derived from the CALUX^®^ estrogen receptor-α (ERα) transactivation assay (Najjar et al., 2024a). The *in vitro* PoD, together with estimated plasma concentrations, were used to determine the highest concentration of daidzein that could be safely used in a body lotion. The Scientific Committee on Consumer Safety (SCCS) published an opinion in 2022 on genistein and daidzein based on traditional safety assessment methods, including legacy *in vivo* data (SCCS, 2022). This type of safety assessment is no longer possible in the EU; therefore, NGRAs endeavor to be protective of human health by ensuring conservative estimations of biological activities, as well as internal concentrations (Dent et al., 2021). In order to be treated as a read-across approach, there were two main assumptions made for this hypothetical case study, firstly, even though daidzein is already considered safe to be used in cosmetics at a certain concentration and that humans can be exposed to it in the diet, it was assumed to be a new chemical and its presence in soya extract omitted from the safety evaluation. Secondly, only *in vitro* data were used for daidzein, while all *in vitro* and legacy *in vivo* data for genistein were considered.

The aim of the work described here was to use a bottom-up approach to build rat and human PBPK models to estimate plasma concentrations of daidzein in the (assumed) absence of *in vivo* PK data. The approach used was based on those previously reported for cosmetics ingredients (Najjar et al., 2021). Once validated, an additional aim was to use the oral rat PBPK model to predict plasma concentrations from the *in vivo* NOAEL for genistein to help set test concentrations in *in vitro* bioassays. Finally, the resulting human PBPK model for daidzein was used to convert the *in vitro* PoD of 33 nM to an external topical dose in a body lotion formulation.

## 2 Methods, parameters and assumptions

**FIGURE 2:**
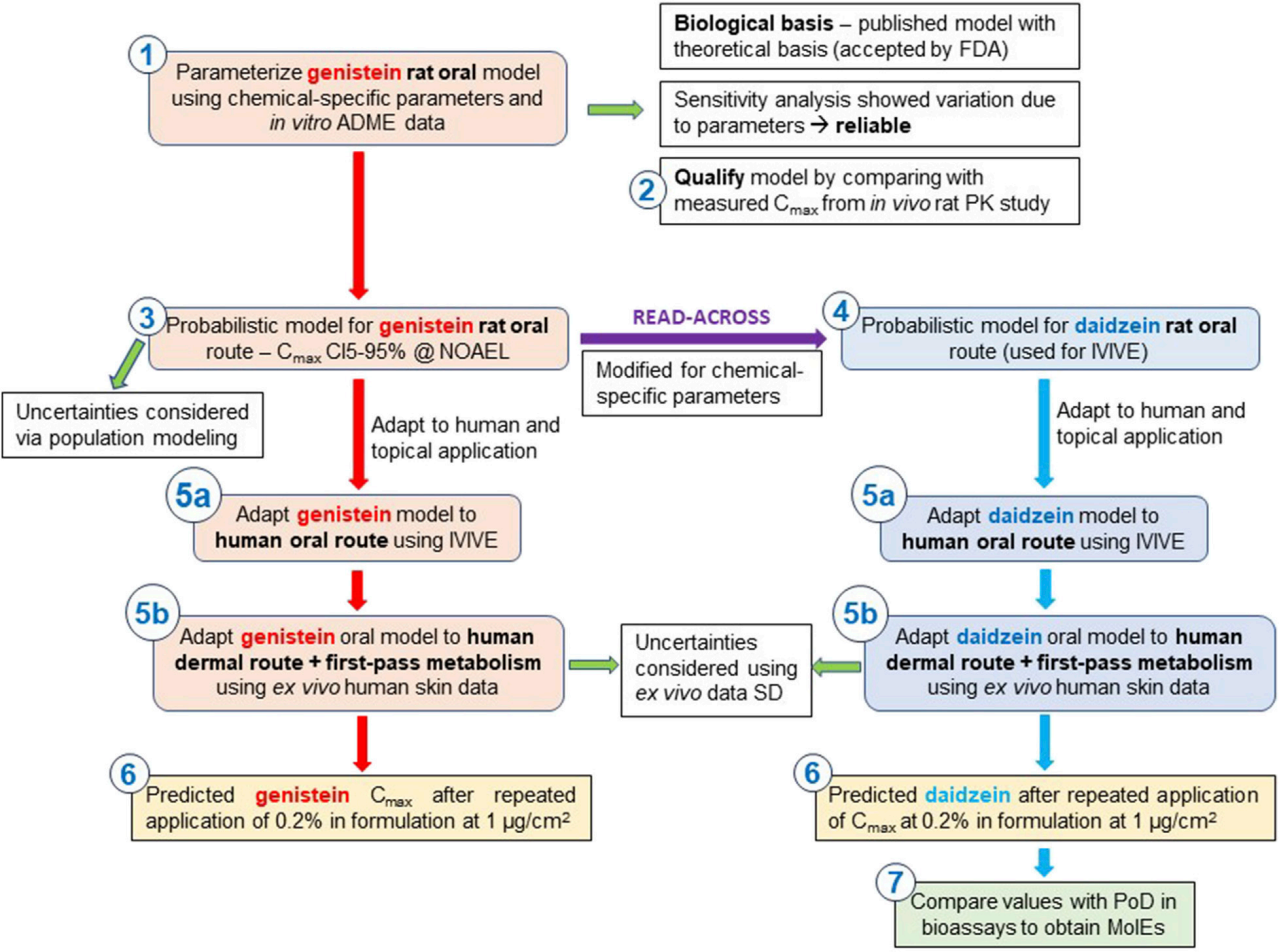
Workflow employed to build two human PBPK models for predicting daidzein and genistein plasma concentrations after topical application and their use in the NGRA case study (SCCS, 2023). Build a mean rat PBPK model for genistein using PK-Sim^®^ and *in vitro* ADME input data (EU, 2009). Validate model by considering the biological basis and conducting a sensitivity analysis. Qualify the model by comparing the predicted C_max_ values with measured values from a legacy *in vivo* PK rat study (Chen et al., 2012). Calculate the CI(5–95)% C_max_ for genistein at its NOAEL in a virtual population of 100 rats and consider uncertainties (Tan et al., 2020). Conduct a read-across: use the PBPK model for genistein a basis for the daidzein oral rat PBPK model and use chemical-specific parameters as input (OECD, 2021b). Adapt the rat models for genistein and daidzein to a human model using relevant physiological parameters (OECD, 2021b). Extend the human model to include a dermal module to be able to mimic topical application of formulations containing genistein or daidzein. Consider uncertainties (OECD, 2021c). Simulate the Cmax of genistein and daidzein for a virtual population of 100 individuals after repeated topical application in a formulation (WHO. Project H, 2010). Compare the C_max_ with the *in vitro* PoD to obtain a so-called “Margin of Internal Exposure” (MoIE) (World Health Organization, 2005) to determine a safe dose of daidzein based on a systemic exposure-based risk assessment.

An overview of the stepwise development of the PBPK models is shown in Figure 2.

### 2.1 Model conceptualization (model structure, mathematical representation)

PK-Sim^®^, OSP Version 10.1 was used (PK-Sim and MoBi (Bayer Technology Services, Leverkusen, Germany: http://open-systemspharmacology.org) (Thelen et al., 2011; Willmann et al., 2007; Willmann et al., 2005; Willmann et al., 2003). An overview of human and rat whole-body PBPK models is provided in Section 1 of the Supplementary Material and model information is reported in the peer review articles in the reference list and on the homepage: https://docs.open-systems-pharmacology.org/. The relevant literature values for anthropometric (height, weight) and physiological information (e.g., blood flows, organ volumes, binding protein concentrations, hematocrit, cardiac output) in adults are incorporated into PK-Sim^®^. The whole-body PBPK model contains an explicit representation of the organs and tissues which have relevant impacts on ADME of a drug (Jones and Rowland-Yeo, 2013). The model assumes the chemical concentration at a steady state in the tissues is in equilibrium with the circulation chemical concentration. Chemical-specific parameters are used to characterize the drug distribution into different tissues in the body (Jones and Rowland-Yeo, 2013).

IVIVE equations are integrated into the software, such that equations automatically convert *in vitro* input values (listed in Table 2) to *in vivo* equivalent values. For example, intrinsic clearance values in hepatocytes, expressed as µL/min/million cells, were converted to predicted *in vivo* hepatic clearance expressed as L/h using the well-stirred model, where the hepatocellularity used was 110 million cells/g, and a value of 67% was taken as the percentage of intracellular space in the liver (a default value in the software).

### 2.2 Step 1: Rat model parameterization using *in vitro* and *in silico* ADME input

The rat physiological parameters included mean body weight of 0.23 kg and an age of 40 weeks and a glomerular filtration rate at 57 mL/min/100 g organ (Davies and Morris, 1993). The physicochemical properties of genistein and daidzein are listed in Table 1. Where possible, measured values were prioritized; however, *in silico* models, e.g., QSARS, were used to predict values in the absence of measured data. The selection of an *in silico* value from different models was according to the applicability domain and prediction probability.

**TABLE 1:** Physicochemical properties of genistein and daidzein.

Property	Genistein		Daidzein	
	Value	References	Value	References
Log P_ow_	3.04^P^	PubChem, (Fabian et al., 2019)	3.24	OPERA (Mansouri et al., 2018)
Boiling point (°C)	555.5	ChemSpider, Biosynth	512	ChemSpider, Chemenu
Melting point (°C)	301.5	ChemSpider, Open Melting Point Dataset	290	ChemSpider, Biosynth
Vapor pressure (mmHg)	1.33 × 10^−9P^	OPERA (Mansouri et al., 2018)	3.46e-10	OPERA (Mansouri et al., 2018)
Molecular weight (g/mol)	270.24	PubChem	254.23	PubChem
Water solubility (mg/mL)	0.12^P^	ADMET Predictor 10 (SimulationsPlus)	0.053	ADMET Predictor 10 (SimulationsPlus)
pKa at 25°C	7.25 ± 0.84	Nan et al. (2014)	7.51 ± 0.07 9.47 ± 0.14	Nan et al. (2014)
Relative Density (g/cm^3^)	1.5 ± 0.1^P^	ChemSpider, (ACD/labs)	1.4 ± 0.1	ChemSpider, (ACD/labs)

Values are measured or predicted (denoted by “P”) according to the reference.

ADME parameters were collected from several sources (Table 2) and the use of these is described below. Since genistein is data-rich, more recent publications (with potentially more sensitive and validated analytical techniques) were considered over older data, e.g., values from Yang, Kulkarni (Yang et al., 2012). However, most measured data were comparable regardless of the date of generation.

**TABLE 2:** Genistein and daidzein ADME parameters used to build the PBPK models.

	Genistein		Daidzein	
Property	Value	References	Value	References
Absorption and bioavailability				
Caco-2 *P*app (A-B) (cm/s)	High; 33.1 ×10^−6^	Papaj et al. (2014), Liu and Hu (2002)	20.3 × 10^−6^	Kawahara et al. (2020)
Caco-2 *P*app (B-A) (cm/s)	33.7 × 10^−6^	Papaj et al. (2014)	Not available	-
Efflux ratio	1.02	Calculated from the ratio of Papp (B-A) to Papp (A-B)	Not available	
*Peff* (cm/s)	1.01 × 10^−4P^	Calculated based on *P*app (A-B)	6.61 × 10^−5^	Calculated based on *P*app (A-B)
Dermal delivery of parent chemical (% of applied dose): *ex vivo* *: Human fresh skin, in formulation at 3* * * *nmol/cm* ^*2*^	7.2 ± 5.4	Géniès et al. (2024)	13.5 ± 7.0	Géniès et al. (2024)
Distribution				
Tissue:plasma partition coefficient	Rodgers and Rowland^P^			
Cellular permeabilities	PK-Sim Standard Model^P^			
Fu, human (%) (measured using rapid equilibrium dialysis)	3.7 ± 0.2; 2.43	Unpublished LRSS data; Wetmore et al., (2015)	3.2; 5.53	Unpublished LRSS data; Wetmore et al. (2015)
Fu, rat (%) (measured using rapid equilibrium dialysis)	2.7; 0.85; 7 ± 0.2; 3.2^P^	Fabian et al., (2019); Honda et al. (2019); Punt et al. (2013); Zhang et al. (2019)	3.15	Honda et al. (2019)
Blood:plasma ratio	0.73	Predicted within PK-Sim	1.08	Predicted within PK-Sim
Metabolism				
Liver (human and rats)	De-toxification (major phase II metabolites) in microsomes and hepatocytes	Bursztyka et al. (2008)	De-toxification (major phase II metabolites) in HepG2 cells	Toro-Funes et al. (2015)
Metabolites formed in *ex vivo* fresh human skin	De-toxification (major phase II metabolites) in fresh human skin	Géniès et al. (2024)	De-toxification (major phase II metabolites) in fresh human skin	Géniès et al. (2024)
Elimination				
ECCS classification	Class 2: metabolism^P^	Varma et al. (2015)	Class 2: metabolism^P^	Varma et al. (2015)
Renal clearance (L/h)	0 (*in vivo*)	Yang et al. (2012)	0	Based on genistein data
Transporters substrate	No (*in vivo* */* *in vitro* (Caco-2))	Yang et al. (2012)	No	Based on genistein data
Hepatic clearance, human (µl/min/10^6^ cells)	18.71 (pooled cryopreserved hepatocytes)	Wetmore et al. (2015)	15.4	Wetmore et al. (2015)
Hepatic clearance, rats (µl/min/10^6^ cells)	68 (rat hepatocytes)	Honda et al. (2019)	31.8	Honda et al. (2019)
Hepatic clearance, rats (ml/min/kg bw)	28.1 ± 4.2 (rat liver microsomes)	Punt et al. (2013)	No data	
Skin first-pass (% of dose metabolized) *in ex-vivo* human fresh skin, in formulation at 3 nmol/cm^2^	70–90	Géniès et al. (2024)	36	Géniès et al. (2024)

#### 2.2.1 Absorption and oral bioavailability

For oral exposure in the rat, the apparent permeability (*P*app) of genistein and daidzein was first estimated according to the Caco-2 assay (Papaj et al., 2014; Kawahara et al., 2020). The efflux ratio (i.e., *P*app(B-A)/*P*app(A-B)) was 1.02, indicating that transporter-mediated efflux genistein is not occurring, i.e., genistein is not actively pumped out of the cells. Based on genistein results, it was assumed that daidzein efflux also does not occur. The permeability data correspond to highly permeable chemicals, which was confirmed for genistein by Liu and Hu, (2002). The effective permeability (*Peff*) in rats was further estimated using a correlation between *Peff* [from an *in-situ* intestinal rat perfusions assay (Lozoya-Agullo et al., 2015)] and measured *P*app values for reference compounds (unpublished LRSS data). The corresponding *Peff* of genistein and daidzein were estimated to be 1.01 × 10^−4^ and 6.61 × 10^−5^ cm/s, respectively, which was used to parameterize the oral bioavailability model in rats.

#### 2.2.2 Distribution

A perfusion-limited kinetic model was implemented to describe the genistein and daidzein kinetics in the body. Several values were reported for the fraction unbound (Fu) of genistein and daidzein in human and rat plasma but all indicated a high extent of plasma binding (Table 2), as reported by others (Coldham and Sauer, 2000). The contribution of transporters to the chemical distribution was not considered in the current PBPK models since the efflux ratio from the Caco-2 assay for genistein was 1.

#### 2.2.3 Metabolism

Genistein is metabolized predominantly in the liver via phase II reactions (to glucuronide and sulfate conjugates), with a minor role of phase I reactions [according to results from incubations with liver microsomes and hepatocytes from human and rats (Bursztyka et al., 2008)]. Daidzein is metabolized in the same manner as genistein, with only phase II metabolites identified (Toro-Funes et al., 2015). *In silico* models i.e., Meteor (Lhasa) and GLORYx (de Bruyn Kops et al., 2021) were implemented to predict the possible metabolites. Meteor Nexus: 3.2.0, Nexus: 2.6.0 used the following prediction method options: Site of Metabolism Scoring (with Molecular Mass Variance), with a Molecular Mass Similarity Threshold of 70, a Scoring filter set at “relative”, and a score threshold of 70. Meteor Nexus and GLORYx use a machine learning-based site of metabolism prediction and combines this with reaction rule sets to predict and rank the structures of metabolites potentially formed by phase 1 and/or phase 2 metabolism. The higher the priority score, the higher the ranking of the metabolite and the more likely it is to be formed. In the current study, GLORYx predicted the highest priority scores and ranking resulted from phase II reactions on the aromatic hydroxyl group (Supplementary Figure S1). This correlated with the results of predictions using Meteor Nexus (Supplementary Figure S2).

#### 2.2.4 Elimination

The Extended Clearance Classification System (ECCS) predicts the major route of clearance from the body using the chemical structure (Varma et al., 2015). Genistein and daidzein are predicted as class 2 chemicals, where metabolism is the major route of elimination. Based on *in vitro* incubations with human hepatocytes, the hepatic clearance of both chemicals is comparable (Table 2). Several clinical studies reported that genistein glucuronides and sulfates, but not free genistein, were recovered in the urine (Yang et al., 2012). Therefore, renal clearance was excluded as an elimination pathway for parent chemicals and set at 0 L/h. By contrast, free genistein was identified in the urine of rats, representing up to 20% of the dose (King et al., 1996). Based on the ECCS prediction, observed clinical data and the efflux ratio of 1 for genistein in Caco-2 cells, the role of transporters in the elimination of the test chemicals was excluded.

### 2.3 Step 2: Comparison of predicted and measured *in vivo* genistein PK in the rat

Genistein PK data from the literature were used to validate the rat PBPK model (Yang et al., 2012). Several criteria were considered; 1) since the *in vitro* data were generated for Sprague Dawley^®^ rats (SD-rats), the relevant *in vivo* data in SD rats were selected for the validation; 2) non-pregnant rats; 3) repeated dose exposure; 4) different dosing regimens of pure and measurable amounts of genistein and 5) measurement of genistein as the analyte (rather than total equivalents of genistein after subjecting samples to conjugate hydrolysis using glucuronidase and/or sulfatase). Based on these criteria, the study conducted by Chen and Bakhiet (2006) was selected, in which the steady-state concentrations of genistein in plasma, liver, and skeletal muscle in SD-rats was measured.

### 2.4 Step 3: Prediction of plasma concentration at the NOAEL in the rat

The final rat probabilistic model was used to simulate plasma concentrations according to the dosing scenario in repeated oral dose toxicity studies in rats from which the NOAEL was derived. The virtual rat publication was also created for 100 individuals around the mean value by scaling the weight from 0.185 to 0.275 kg. A population of 100 individuals was considered to adequately consider subject variation while being manageable with regards to computational resources. The CI5-95% C_max_ values were predicted for the genistein NOAEL dose of 0.3 mg/kg/day, which was considered by the SCCS to be the most relevant PoD (SCCS, 2022).

### 2.5 Step 4: Use chemical read-across to build the oral rat PBPK model for daidzein

The structures of daidzein and genistein differ by only one hydroxy group (as shown in Figure 1). Their physicochemical properties (shown in Table 1) and ADME profiles (reported in Table 2) are also comparable. The use of genistein as a source chemical for daidzein was also supported by Najjar et al., (2024a), who compared the quality of different potential analogues using ToxGPS software [developed by Yang et al. (2023)]. The analogue quality considers chemical similarities using MACCS and ToxPrint Fingerprints, Chemotype profiles, molecular properties, including quantum mechanical parameters, and Skyline profiles. This evaluation indicated that the closest analogue to daidzein was genistein. Thus, the PBPK model for genistein was extrapolated to build a rat PBPK model for daidzein. For this, the physicochemical properties of daidzein listed in Table 1 were used as input for the oral rat PBPK model. ADME parameters for daidzein are listed in Table 2.

### 2.6 Step 5: Adapt rat models to build human models including the dermal route

#### 2.6.1 Step 5a: Human model parameterization

A human PBPK model was developed using a population of individuals incorporated in PK-Sim^®^, and IVIVE using *in vitro* ADME data for genistein and daidzein (Table 2). The rat physiological parameters were changed to those for a European human individual characterized according to parameters representing the mean values of age (30 years), body weight (60 kg), height (163 cm), BMI (22.58 kg/m^2^), body surface area (1.65 m^2^) and GFR (107.44 mL/min) (Davies and Morris, 1993). Population PBPK modeling was applied to 100 individuals within the European population to consider individual anatomy and physiology variations. The age-based population was generated by scaling the age from 16 to 70, where the body weight ranged from 45 to 100 kg. The corresponding physiology and anatomy parameters were dependently ranged (Supplementary Figure S3 displays the range and counts of age and weight of the human individuals). The confidence-interval CI (5–95)% was estimated for the PK parameters of the designed population.

#### 2.6.2 Step 5b: *In silico* human dermal modules

The oral route was not relevant for the human PBPK model, in which dermal exposure was the only route of exposure. Therefore, the human PBPK model was extended to include a dermal module for topical application, based on a skin permeation model published by Dancik et al. (2013). The skin model was built in MoBi (Bayer Technology Services, Leverkusen, Germany). The skin permeation model has several compartments: air, surface pool, vehicle, skin, and *in vivo* link compartment. The skin compartment is a one-dimensional multilayered slab into which an applied chemical passively diffuses. Each slab layer corresponds to a skin layer (stratum corneum, epidermis, and dermis) and derives its diffusion parameters from the layer’s physical and chemical properties. Dermal clearance in *in vivo* simulations of the chemical undergoes passive transport from all sub-layers of the skin layers into the bloodstream. The chemical diffusion through the skin sub-layers is numerically computed using the methods of finite differences and Fick´s law ((Fick, 1995) which models the one-dimensional diffusive flux over a spatial position, see Equation 1 below).

Fick’s first law:

$$J_{\max}=\frac{D}{h}K_{SC/w}C_{w}^{sat}$$ (1)

(Bury et al., 2021).

J_max_ (ng/cm^2^/h) defines the maximum amount of a chemical that can penetrate the skin, D (cm/h) is the diffusion coefficient of the chemical for the stratum corneum (SC), h is the thickness of the SC (µm), and $K_{SC/w}$ the partition coefficient between SC and water (no units).

A metabolism reaction was incorporated into the dermal model to account for metabolism, which was considered a first-order model in the skin living layers of the dermis and epidermis. The metabolism rate was initially predicted using *in silico* QSARs; however, the metabolism rate was adjusted using *ex vivo* skin data (Géniès et al., 2024) regarding skin first-pass metabolism in the viable skin layers (dermis and epidermis). Thus the % of the dose undergoing metabolism was 70%–90% of genistein and 36% for daidzein (Table 2).

#### 2.6.3 Parameterization of the dermal module

The dermal models for genistein and daidzein were parametrized to mimic the observed amounts absorbed in an *ex vivo* study, in which a body lotion containing genistein or daidzein was applied to *ex vivo* human skin (Géniès et al., 2024). The *ex vivo* study results (Table 2) were implemented to calibrate the default dermal model in human, where genistein and daidzein are non-volatile neutral organic chemicals, without a pharmacophore. The model was based on fully hydrated full thickness skin at 1,500 µM divided into three sub-layers (stratum corneum, epidermis and dermis with a thickness of 43 μm, 60 μm, and 1,400 μm, respectively). The skin surface temperature was set at 30°C and the wind velocity was set at 16.8 cm/s.

Information about the skin metabolism were retrieved from incubations of genistein and daidzein applied to the surface of *ex vivo* fresh human skin (Géniès et al., 2024) to estimate the first-pass metabolism in the skin after topical application. Mono-glucuronide and mono-sulfate conjugates of genistein were detected in the medium 18 h after application, with the glucuronide being the major metabolite. After application of 3 nmol/cm^2^ of genistein in ethanol or body lotion formulation to *ex vivo* fresh human skin, ∼80% of the dose was recovered as metabolites in the skin and medium. The metabolism of 3 nmol/cm^2^ daidzein in ethanol or body lotion formulation applied to the skin is lower, with around 40%–47% of the dose recovered as metabolites in the skin and medium. For both chemicals, there was a linear correlation between the dose and the formation of the sulfate conjugate over 3–30 cm^2^, indicating that the sulfotransferases involved in its metabolism were not saturated. There was a small deviation from linearity for the glucuronide conjugate, indicating that the UDP-glucuronosyltransferase are saturated ≥30 nmol/cm^2^ (Géniès et al., 2024). The skin metabolism was incorporated in the dermal model to account for the first-pass metabolism at 70% of dermally penetrated genistein and 36% of dermally penetrated daidzein.

### 2.7 Predictions using final rat and human PBPK models

For Step 2, the final oral rat model was used to simulate a repeated dose toxicity study for oral exposure.

For Step 6, the final human PBPK models were used to simulate repeated dermal exposure of genistein and daidzein present in a body lotion or face cream. The exposure scenarios for both models are summarized in Table 3.

**TABLE 3:** Exposure scenarios of genistein and daidzein in the rat and human models.

(A) Rat	
Administration route	Oral
Dose	@ NOAEL mg/kg/day
Dose frequency	Repeated
Dose per day	One per day for up to 5 weeks
Formulations (as powder of high-genistein isoflavone diets)	Suspension- Weibull
	Time to dissolve 50% = 250 min (default)
	Lag time 0 (default) Shape = 0.92 (parabolic), (default)
(B) Human	
Administration route	Dermal
Amount of chemical applied	1 µg/cm^2^
Amount of face cream applied	1.54 g/day
Amount of body lotion applied	7.82 g/day
Face surface area	565 cm^2^
Whole-body surface area	15,670 cm^2^
Dose per day	one per day for 7 days.

Reverse dosimetry using the rat oral model was used to convert the *in vitro* PoD of 33 nM (a) to the bioequivalent oral dose of daidzein in rats (Najjar et al., 2024a) and (b) the concentration of daidzein in a body lotion or face cream topically applied to humans using the human model. The external doses were identified by modifying the dose applied until the target C_max_ values were obtained.

### 2.8 Sensitivity and uncertainty analysis

A local sensitivity analysis of the PBPK models was conducted according to organism/chemical-specific input parameters using PK-sim. This identifies a set of variables that impact the estimated PK. The sensitivity analysis was conducted in three steps, with a variation range of 10%/step. The sensitivity for the PK Parameter = PKj to an input parameter = [ pi ] was calculated as the ratio of the relative change of that PK Parameter [ = (ΔPKj)/PKj ] and the relative variation of the input parameter [ = (Δpi)/pi ] according to Equation 2:

$$S_{i,j}=\frac{{\Delta PK}_{j}}{{\Delta p}_{i}}\;.\;\frac{p_{i}}{{PK}_{j}}$$ (2)

The sensitivities are dimensionless quantities calculated as the average of several sensitivities based on different variations. The parameters were then ranked to determine which had the greatest influence on the output. With the calculated absolute values of a normalized coefficient, sensitivity are classified as (OECD, 2021b):

− High:≥0.5

− Medium:0.2≤medium< 0.5

− Low:0.1≤low< 0.2

An uncertainty analysis of the PK parameters was addressed by the confidence interval CI (5–95)%, by which the influencing parameters were ranged by the standard deviation around the mean of the chemical-specific parameters. The level of uncertainty per parameter was estimated based on the equation published by Najjar et al. (2024b) for the considered PK parameters. Uncertainty analysis results are categorized as a high uncertainty (value could be a factor of 2 or higher); a medium uncertainty (value could be a factor between 0.3 and 2) or a low uncertainty (value could be a factor of 0.3 or lower) (OECD, 2021b).

## 3 Results

### 3.1 Rat-PBPK model

#### 3.1.1 Comparison of predicted and measured genistein plasma concentrations

**FIGURE 3:**
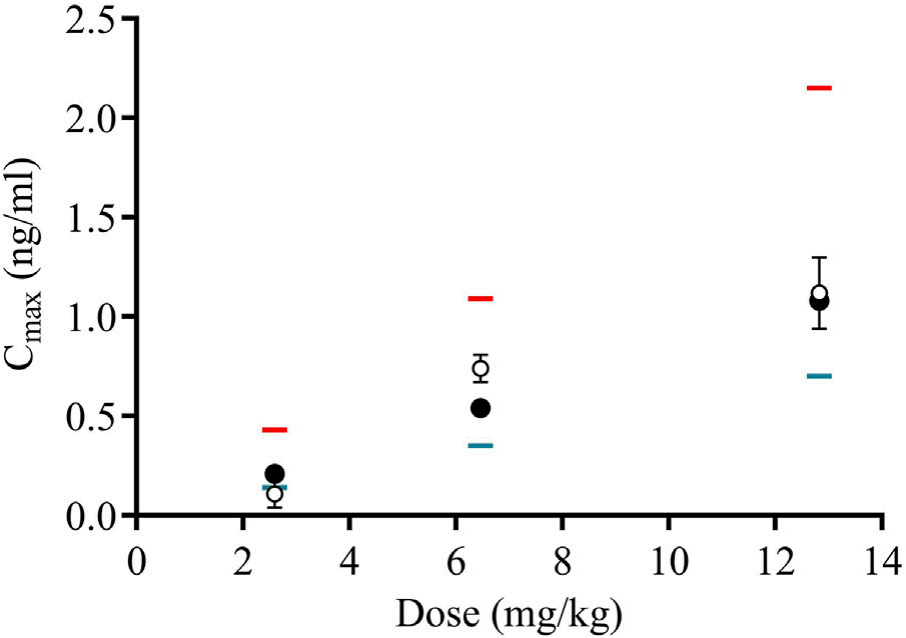
Correlation between observed and predicted maximum plasma concentrations (C_max_) of genistein in rats. Values for *in vivo* observed concentrations after oral administration (open circles with SD error bars) were from Chen and Bakhiet (Chen and Bakhiet, 2006). The predicted values are shown as the mean (black circles), CI95% (red lines) and CI5% (blue lines).

The developed rat PBPK model for genistein was evaluated according to its ability to reproduce *in vivo* PK data in rats treated with 3 oral doses of genistein (Chen and Bakhiet, 2006). The predicted C_max_ values correlated well with the observed values, with an *R* ^2^ of 0.98 for the mean predicted values (Figure 3). The measured C_max_ values were within the predicted CI5% and CI95% values. Table 4 shows the fold errors of the prediction of the C_max_ values. These were all within 2-fold of the measured C_max_ values, with the exception of the CI95% value for the 2.59 mg/kg dose (3.9-fold higher than the mean C_max_ value).

**TABLE 4:** Fold error (predicted:measured) values for the simulated plasma C_max_ concentrations of genistein in rat plasma.

Dose (mg/kg)	Fold error (predicted:measured Cmax)		
	CI 95%	Mean	CI 5%
2.59	3.90	1.90	1.27
6.46	1.47	0.73	0.47
12.82	1.92	0.96	0.63

Values for *in vivo* observed concentrations of genistein after oral administration were from Chen and Bakhiet (Chen and Bakhiet, 2006). The predicted values compared were the mean, CI95% and CI5%.

#### 3.1.2 Uncertainty and sensitivity analyses

The C_max_ was sensitive to several parameters related to the organism, chemical-specific, and formulation parameters (Supplementary Figure S4). The uncertainty in the physiological and anatomical parameters was considered as a part of the population PBPK modeling, where they were scaled based on the mean individual. There were several chemical-specific inputs which influenced the estimated C_max_, including the hepatic clearance and lipophilicity. Therefore, a range of these parameters based on the *in vitro* reported values was considered to estimate the CI 5%-95% C_max_. The C_max_ was also sensitive to formulation parameters used in the rat study, including dissolution time and shape. As these were implemented as default values, ranges of these values were defined using a default coefficient of variation at 30% and a normal distribution (Clewell and Clewell, 2008).

#### 3.1.3 Estimation of plasma concentrations at genistein NOAEL

Since the PBPK model for genistein was able to reproduce the observed C_max_ values (Figure 3), a probabilistic PBPK model was developed to estimate the range of C_max_ values within a virtual population of rats to account for the anatomy and physiology variations and the uncertainty of the chemical-specific parameters that have an impact on the final estimated C_max_. This allowed an estimation of the C_max_ of genistein associated with its NOAEL in repeated dose studies. After converting the model using *in vitro* ADME data for daidzein, both rat PBPK models were used to simulate concentration-time profiles of genistein and daidzein in rat plasma after repeated oral exposure over 7 days to the NOAEL of genistein of 0.3 mg/kg/day (Supplementary Figure S5). The estimated total and unbound C_max_ values for daidzein in rats were higher than those for genistein (Table 5).

**TABLE 5:** Simulated plasma concentrations of genistein and daidzein in rat plasma after repeated oral exposure at 0.3 mg/kg/day over 7 days using probabilistic PBPK models for both chemicals.

Parameter	Genistein	Daidzein
C_max, total_ (nM)	24.1	45.17
C_max, total_ (CI5–95%) (nM)	12.4–61.5	17.14–135.9
C_max,fu_ (nM)	0.7	1.33
C_max,fu_ (CI5–95%) (nM)	0.38–1.37	0.72–2.61

### 3.2 Human-PBPK models

#### 3.2.1 Route extrapolation - simulations using fresh human skin

**FIGURE 4:**
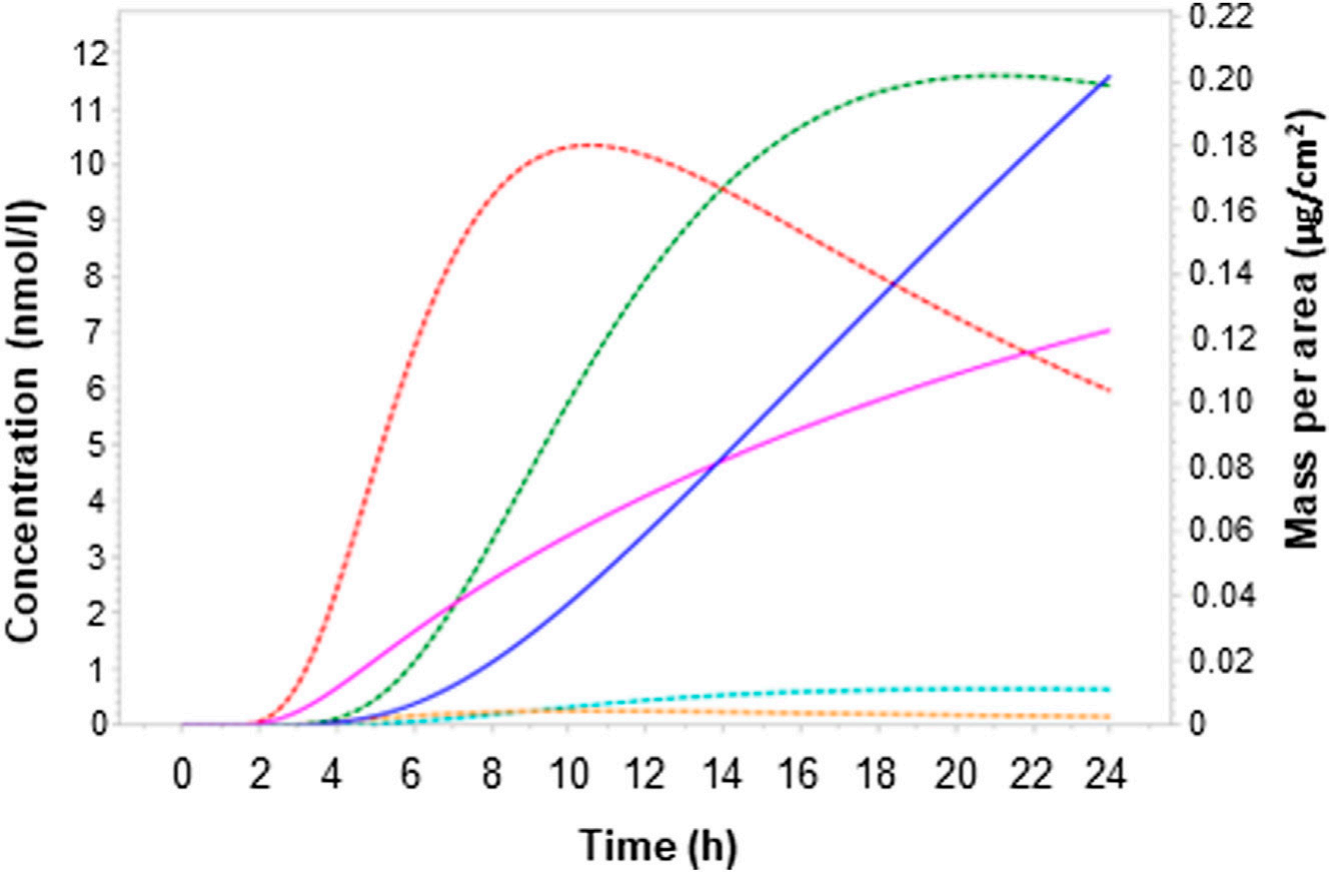
Simulated concentration-time profile and dermal delivery of daidzein and genistein in human after a single topical exposure as body lotion (0.2% of genistein/daidzein in the formulation at 0.5 mg/cm^2^ and equivalent to 1 μg/cm^2^ of daidzein and genistein) to fresh viable skin with first-pass metabolism. Dermal delivery is the amount of chemical considered to by systemically available and is calculated as the percentage of the applied dose recovered in the epidermis, dermis and receptor fluid (SCCS, 2023). Dark blue solid lines represent the dermal delivery of daidzein; dotted green lines represent total plasma concentrations of daidzein; light blue lines represent unbound plasma concentrations of daidzein. Pink solid lines represent the dermal delivery of genistein; dotted red lines represent total plasma concentrations of genistein; dotted orange lines represent unbound plasma concentrations of genistein.

The dermal models for genistein and daidzein were refined to mimic the reported penetration in fresh viable human skin over 24 h (Figure 4). The refinement was conducted using the low exposure at 1 μg/cm^2^, which is comparable to the exposure of 3 nmol/cm^2^ tested in *ex vivo* experiments. The resulting predicted plasma concentrations after topical application indicated that the different rates of first-pass metabolism of the chemicals impacted the relative concentrations of genistein and daidzein, whereby less extensive first-pass metabolism of daidzein meant that total and unbound C_max_ values were higher for daidzein than genistein (Table 6).

**TABLE 6:** Comparison of simulated concentrations and dermal delivery of daidzein and genistein in human after a single topical exposure as body lotion (0.2% of genistein/daidzein in the formulation at 0.5 mg/cm^2^ and equivalent to 1 μg/cm^2^ of daidzein and genistein) using fresh viable skin with first-pass metabolism.

	Genistein	Daidzein
Applied dose (µg/cm^2^)	1	1
Dermal delivery over 24 h (µg/cm^2^)	0.12	0.20
Dermal delivery over 24 h (% Applied dose)	12	20
Predicted plasma C_max_ (nM)	10.4	11.6
Predicted plasma C_max,fu_ (nM)	0.32	0.65

Dermal delivery is the amount of chemical considered to by systemically available and is calculated as the percentage of the applied dose recovered in the epidermis, dermis and receptor fluid (SCCS, 2023).

#### 3.2.2 Uncertainty and sensitivity analyses

The uncertainty calculated in the current study covers both true uncertainty (chemical-specific parameters) and variability (population variability). The sensitivity analysis identified several influencing chemical-specific parameters (fu, hepatic clearance, skin metabolism rate) (Supplementary Figure S6). The skin permeation model was optimized to deliver the measured amount in the *ex vivo* experiments, which was used to parametrize skin metabolism within the skin module. Thus, the physiological and anatomical parameters influencing the outcome were considered within the population modeling. This allows estimating the CI 5%-95% C_max_ within the population, including the uncertainty of the chemical-specific parameters.

#### 3.2.3 Estimation of plasma concentrations after topical application of genistein and daidzein

**FIGURE 5:**
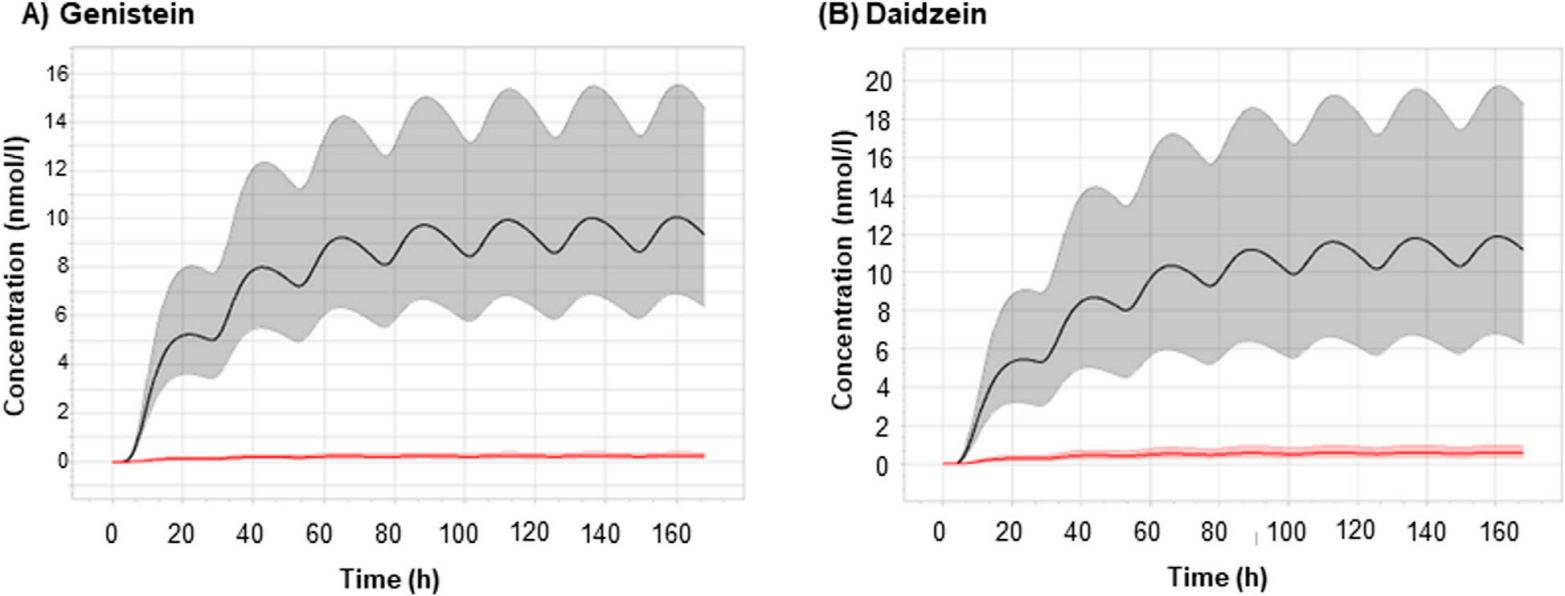
Simulations using probabilistic PBPK modelling of the concentration-time profile and dermal delivery of **(A)** genistein and **(B)** daidzein in human after repeated topical exposure as body lotion (0.2%) at 0.5 mg/cm^2^ (1 μg/cm^2^ of daidzein and genistein). Dermal delivery is the amount of chemical considered to by systemically available and is calculated as the percentage of the applied dose recovered in the epidermis, dermis and receptor fluid (SCCS, 2023). The solid black lines represent the geometric mean total plasma concentrations, and the solid red lines represent the geometric mean unbound plasma concentrations. The regions shaded in gray represent the range of total plasma concentrations between the 5^th^ and 95^th^ percentiles. The regions shaded in pink represent the range of unbound plasma concentrations between the 5^th^ and 95^th^ percentiles.

The probabilistic models were used to estimate the C_max_ of genistein and daidzein within a human population and considering the uncertainty of the chemical-specific parameters, after repeated dermal exposure of a body lotion (0.2%) at 1 μg/cm^2^ (Figure 5). The resulting predicted plasma concentrations indicated that the total and unbound concentrations of parent chemicals were higher for daidzein than genistein (Table 7).

**TABLE 7:** Simulations using probabilistic PBPK modelling of plasma concentrations of daidzein and genistein in human after repeated topical exposure as body lotion (0.2%) at 0.5 mg/cm^2^ (1 μg/cm^2^ of daidzein and genistein) (see Figure 5 for the profiles).

	C_max, total_ (nM) mean	C_max, total_ (nM) CI (5–96)%	C_max,fu_ (nM) mean	C_max,fu_ (nM) CI (5–96)%
Genistein	10.1	6.9–15.5	0.3	0.2–0.4
Daidzein	11.9	6.8–19.7	0.6	0.4–1.0

## 4 Discussion

We describe the workflow by which two human PBPK models for predicting daidzein and genistein plasma concentrations after topical application in a formulation were developed using *in vitro* ADME data for human skin and legacy *in vivo* PK data for genistein in rats. The development of the PBPK models (summarized in Table 2), is similar to the approach used by others who built a PBPK model with a dermal module to estimate the plasma concentrations of homosalate according to consumer use in a sunscreen (Najjar et al., 2021). The general concept of building the rat and human PBPK models was according to that described by Kuepfer et al. (2016) and considered the workflow proposed by OECD guidance (OECD, 2021b). An alternative approach is to build a human oral PBPK model based on human clinical data for genistein and then adapt it for topical application by including a dermal module, which would have a higher level of confidence than the approach used here. However, studies reporting genistein PK profiles in humans after oral consumption used glucuronidase and/or sulfatase to hydrolyze the genistein conjugates to genistein before analyzing the samples (Anupongsanugool et al., 2005; Fanti et al., 1999; Joshi et al., 2007; King and Bursill, 1998; Setchell et al., 2003); therefore, plasma analyte concentrations were not specific to genistein but to total equivalents of genistein i.e., parent and glucuronide and sulfate metabolites. This means that it is not possible to make a direct comparison between clinical concentrations and predicted genistein concentrations using the human model built here (which predicts concentrations of genistein only based on parent compound depletion). It should be noted that for the qualification of the oral rat PBPK model, the measured values were of genistein only, which is presumably why there was a good correlation between predicted and measured Cmax values. Additional uncertainties in the clinical studies are with respect to the different sources of genistein consumed (e.g., “isoflavone-rich soy protein isolate”, commercial soy extract capsules and debittered soy flour), reporting of the amount of genistein and its glycoside form, genistin, present, and the impact of co-administered food or drink before and during the blood sampling. It should also be noted that clinical data are very uncommon for cosmetics ingredients (unless they also happen to be components of the diet, as in the case of genistein and daidzein), and our aim was to develop an approach which is applicable for cosmetics for which clinical data for analogues are unavailable. Another reason for basing the development of the human model on the rat oral data was due to the aim of the NGRA case study, which was to use a read-across approach. For this, legacy data for genistein was used to evaluate the safety of daidzein. Genistein toxicity data were from rat studies, from which the NOAEL was derived and converted to a Cmax. Since the model performed well in rats, it was directly extrapolated to the human dermal model.

### 4.1 Validation and level of confidence

Several aspects were used to evaluate the validity of the developed models. These are described in detail in the WHO guidelines published in 2010 (WHO. Project H, 2010) and (OECD, 2021b; OECD, 2021c). The models have reasonable biological basis, which does not violate what is known about the physiology of the modeled organism. The genistein PBPK model was validated using *in vivo* PK data, whereby it was able to reproduce the observed C_max_ values for genistein in the rat. There was only a small fold error in the predicted mean values from the measured values, with fold errors of less than 2-fold. This indicates that the model is valid according to the WHO guidelines, which states that (along with other criteria) a model can be validated if the ratio is < 2 (WHO, 2010).

Genistein PK data were implemented to verify the predictive ability of the developed PBPK model for daidzein. Based on OECD 2021 guidance, the predictive performance of the genistein PBPK model can be extended to the daidzein PBPK model. Moreover, the sensitivity analysis, population variability, and parameter uncertainty were considered to estimate the CI 5%-95%, within the population modeling, and to support the robustness of the model. Thus, the developed PBPK models were assigned a moderate level of confidence based on the OECD 2021 guidance, where local sensitivity analysis was performed instead of global analysis.

### 4.2 Mimicking effects of first-pass metabolism in the skin

*Ex vivo* experiments on fresh skin were used to calibrate the dermal model in the human PBPK model. These data proved to be important in the estimation of the plasma concentrations of the two chemicals since both were extensively metabolized as they penetrated fresh viable human skin. The different rates of first-pass metabolism of the two chemicals impacted their relative plasma concentrations, whereby C_max_ values were higher for daidzein than genistein in fresh viable skin. This was important in the NGRA since the metabolites of both chemicals were revealed to exhibit low (if any) bioactivity (Najjar et al., 2024a). This meant that if the amount of parent chemical entering the systemic circulation was based on data from incubations with frozen human skin, there would have been an overestimation of the internal exposure of the parent chemical, especially of genistein (for which the penetration of total parent chemical in frozen skin was ∼13-fold higher than in fresh skin (Géniès et al., 2024). While this could be interpreted as a conservative approach, it is also less realistic given the large difference in internal exposure, which could make a large impact on the MoIE.

Another important aspect of the *ex vivo* experiments is that they mimicked the body lotion formulation used by consumers. In the same study by Géniès et al. (2024) the dermal deliveries of 3 nmol/cm^2^ genistein and daidzein were much higher when they were applied in 100% ethanol to fresh human skin (83% compared to 45% of the applied dose for genistein in ethanol and formulation, respectively, and 65% compared to 25% of the applied dose for daidzein in ethanol and formulation, respectively). Therefore, the dermal modules of the PBPK models were relevant to the application scenario used in the read-across case study.

### 4.3 Use of PBPK modeling in an NGRA case study

**FIGURE 6:**
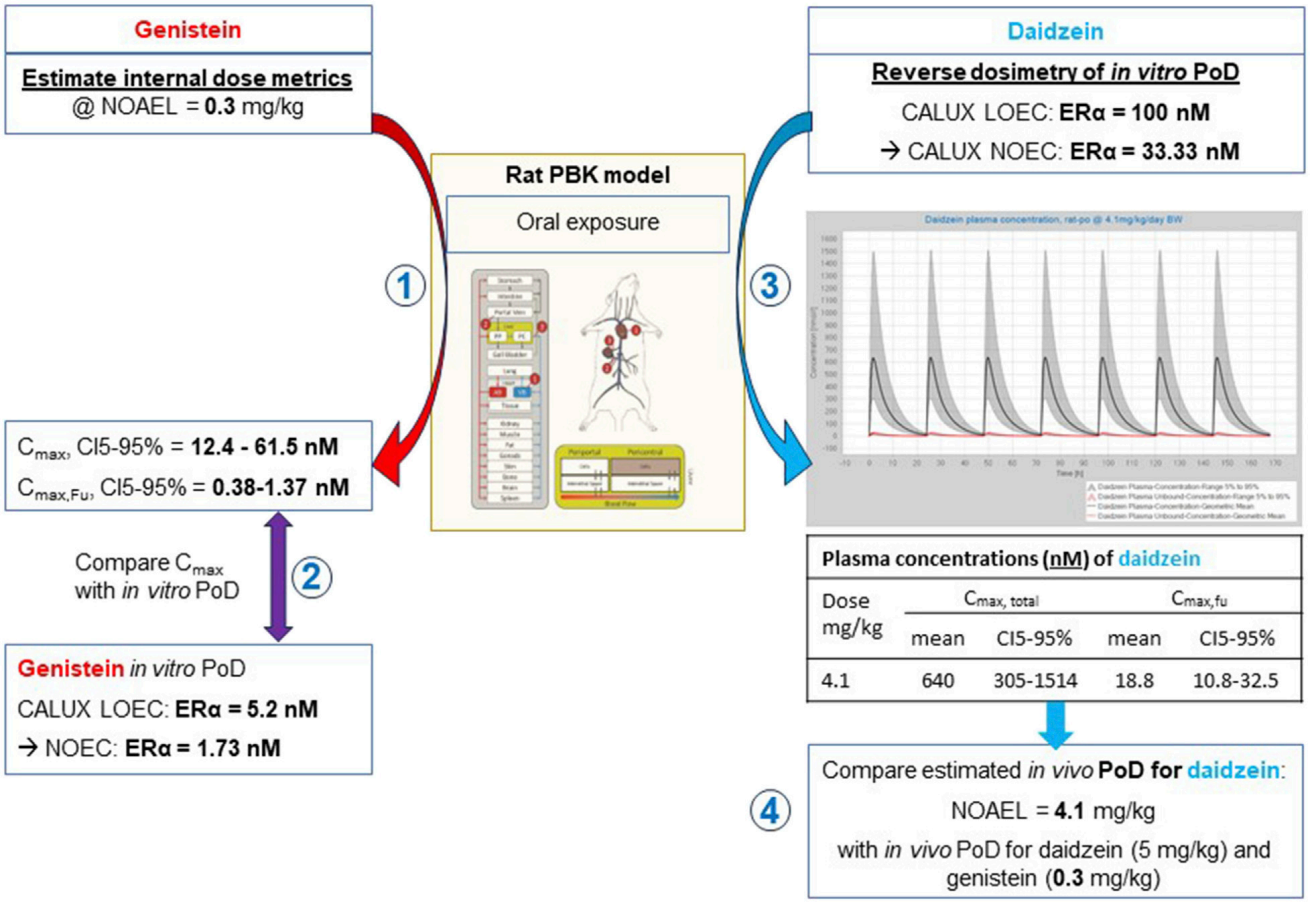
An overview of the development of the PBK models, together with the associated assessments for model qualification, estimation of plasma concentrations after topical application and the derivation of the MoIE (SCCS, 2023). Conversion of a NOAEL of genistein (0.3 mg/kg/day) in rats to an internal plasma concentration (EU, 2009). Predicted concentrations were compared with the *in vitro* value equivalent to a NOAEL, i.e., an *in vitro* PoD (Chen et al., 2012). Reverse-dosimetry of the *in vitro* PoD to estimate the equivalent external oral dose of daidzein to rats (Tan et al., 2020). The estimated external *in vivo* PoD of 4.1 mg/kg correlates with the *in vivo* PoD of 5 mg/kg identified by the SCCS (SCCS, 2022) and the known lower potency of daidzein compared to genistein [the *in vivo* PoD is 0.3 mg/kg (SCCS, 2022)].

In the read-across case study, the PBPK models were used to convert a NOAEL of genistein (0.3 mg/kg/day) in a repeated dose toxicity assay in rats to an internal plasma concentration (Figure 6). This was then used to set the concentrations tested in a range of *in vitro* bioassays. The result of the simulation was also used to support the use of the rat oral PBPK model in the safety assessment. The mean C_max, total_ was predicted to be 24.1 nM, (CI5-95% ranging from 12.4 to 61.5 nM) and the mean C_max,fu_ was predicted to be 0.7 nM (CI5-95% ranging from 0.38 to 1.37 nM). These concentrations were compared with the *in vitro* value equivalent to a NOAEL, i.e., an *in vitro* PoD. This was derived from the CALUX^®^ ERα transactivation assay (van der Burg et al., 2015), in which the “Lowest Observed Effect Concentration” (LOEC) for genistein was 5.2 nM. To estimate the PoD, i.e., the No Observed Effect Concentration (NOEC), the LOEC was divided by a factor of 3 [recommended by Yang et al., (2017)], resulting in a NOEC of 1.73 nM. This *in vitro* NOEC was ∼14-fold lower than the equivalent *in vivo* NOAEL C_max, total_ (thus indicating the greater conservatism of the *in vitro* NGRA than the PoD used in a traditional risk assessment) but was in the same order of magnitude (nM range), indicting the PBPK model could predict relevant plasma concentrations. The *in vitro* NOEC of 1.73 nM was, however, similar to the CI95 *in vivo* NOAEL unbound plasma concentration of 1.37 nM. This indicates that the fraction unbound of genistein (the active form in blood) could be used to represent relevant internal dose metrics to estimate the external dose of the *in vitro* PoD for genistein and daidzein, while the total concentration represents a more conservative internal dose metrics.

An additional evaluation was made to add confidence to the use of the rat oral PBPK model, although this is not part of the NGRA case study [since the comparison data for daidzein were from an *in vivo* rat study). In this evaluation, reverse-dosimetry of the *in vitro* PoD was conducted to estimate the equivalent external oral dose of daidzein to rats. The LOEC for daidzein in the ERα transactivation assay was 100 nM, which results in a NOEC of 33.33 nM when converted using a factor of 3. When this was converted to an external dose using reverse-dosimetry in the rat PBPK model, this resulted in an estimated external *in vivo* PoD of 4.1 mg/kg. This correlated very well with the *in vivo* PoD of 5 mg/kg identified by the SCCS (SCCS, 2022) and was in accordance with the known lower potency of daidzein compared to genistein (SCCS, 2022)].

## 5 Conclusion

We describe a bottom-up approach to develop human PBPK models for estimating the plasma concentrations of genistein and daidzein after topical application in a body lotion formulation. The oral rat PBPK model was used early in the case study to predict plasma concentrations from the *in vivo* NOAEL for genistein to set test concentrations in *in vitro* bioassays. The final human PBPK model for genistein was used to compare the predicted plasma concentration with the *in vitro* PoD (i.e., the MoIE), and the daidzein model was used to perform reverse dosimetry to convert the *in vitro* PoD for daidzein to an external dose.

Intrinsic hepatic clearance and plasma Fu values were identified as sensitive parameters impacting the predicted C_max_ values. The initial oral rat model for genistein was qualified by comparing predicted values with measured *in vivo* PK values. This adds confidence to the overall capacity of the models to accurately reflect internal exposures. Indeed, when the *in vivo* NOAEL for genistein from a reproduction toxicology study was converted to a plasma concentration, the mean and range of values for C_max_, especially the unbound fraction, correlated very well with the *in vitro* PoD derived from an assay relevant to reproduction, namely, agonism of ERα. An important aspect of the development of the dermal module for the human whole body PBPK model was the implementation of first-pass metabolism, which was extensive for both chemicals. If values for skin penetration from frozen skin assays had been used, the plasma concentrations could have been over-estimated by a considerable amount, especially for genistein. While this could be considered conservative, it would not be accurate.

In conclusion, we have developed two PBPK models for genistein and daidzein which were used as a central component of an NGRA read-across case study. This type of case study will help to gain regulatory confidence in the use of PBPK models, especially for cosmetic ingredients, for which animal-free alternatives are increasingly demanded worldwide.

## Data Availability

The raw data supporting the conclusions of this article will be made available by the authors, without undue reservation.

## Abbreviations

ADME, Absorption, distribution, metabolism, and elimination; Cmax, Maximal plasma concentration; Cls, Systemic clearance; C0, Initial plasma concentration; IV, Intravenous; IVIVE, *In vitro* to *in vivo* extrapolation; MoIE, Margin of internal exposure; PBPK, Physiologically based Pharmacokinetic; PK, Pharmacokinetic; P-gp, P-glycoprotein; PoD, Point of departure; Q, Cumulative mass permeation over time; t1/2, Half-life; Vdss, Volume of distribution at steady state; Tmax, Time to reach Cmax.
