# Supplementary material for: Development and validation of PBPK models for genistein and daidzein for use in a next-generation risk assessment
Source: Front Pharmacol. 2024 Oct 3;15:1421650. doi: 10.3389/fphar.2024.1421650 (PMC11483610; doi:10.3389/fphar.2024.1421650)
Supplement: Supplementary file 1 [file DataSheet1.PDF]

Supplementary Materials

1 Overview of human and rat whole-body PBPK models

The description of the standard model for small molecules in PK-Sim is available at the link: https://docs.open-systems-pharmacology.org/working-with-pk-sim/pk-sim-documentation/pk-sim-simulations#model-settings. Each organ is subdivided into four sub-compartments for small molecules representing plasma, blood cells, interstitial space, and intracellular space. The model sub-compartments describe the organ physiology and anatomy that differs from one organ to another. These parameters were used as default values, which were stored in the PK-Sim database. The arterial and venous blood compartments link the organs/tissues (**Supplementary Figure S1**). Each of the organs/tissues is characterized by several parameters representing associated blood flow, volume, tissue-partition coefficient, and permeability (Kuepfer et al., 2016). The physiologic parameters (organ volumes, organs composition, vascular and cellular space of the organs, surface area, expression levels, blood flow rates, and hematocrit) for the different species were retrieved from the knowledge available a priori on the anatomy and physiology (Davies & Morris, 1993; Edginton et al., 2006; Mordenti, 1986). Such properties vary depending on the species and populations (Kuepfer et al., 2016). The tissues are typically described as either perfusion rate-limited or permeability rate-limited kinetics (Jones et al., 2011; Nestorov, 2007). Generic PBPK models of small molecule drugs assume perfusion rate-limited kinetics, with the liver and kidney being the only clearance sites (Chen et al., 2012; Sinha et al., 2012). The perfusion rate/ blood flow limited kinetics within the organs tend to occur for small lipophilic molecules, where the blood flow becomes the limiting process (Jones & Rowland-Yeo, 2013).

2 Supplementary tables and figures

Supplementary Table S1. Characteristics and biometric data of European individuals (female) used in PBPK modeling (Valentin, 2002).

Age (year/s)	30.00
Weight (kg)	60.00
Height (cm)	163.00
BMI: Body Mass Index (kg/m²)	22.58
body surface area (BSA) (m²)	1.65
Glomerular filtration rate (GFR) (ml/min)	107.44

Supplementary Figure S1. Possible metabolites (with priority score) of (A) genistein and (B) daidzein, predicted by GLORYx (de Bruyn Kops et al., 2021). The green box indicates those metabolites identified by *in vitro* experiments. The higher the priority score, the more likely the metabolite is to be formed.

(A) Genistein

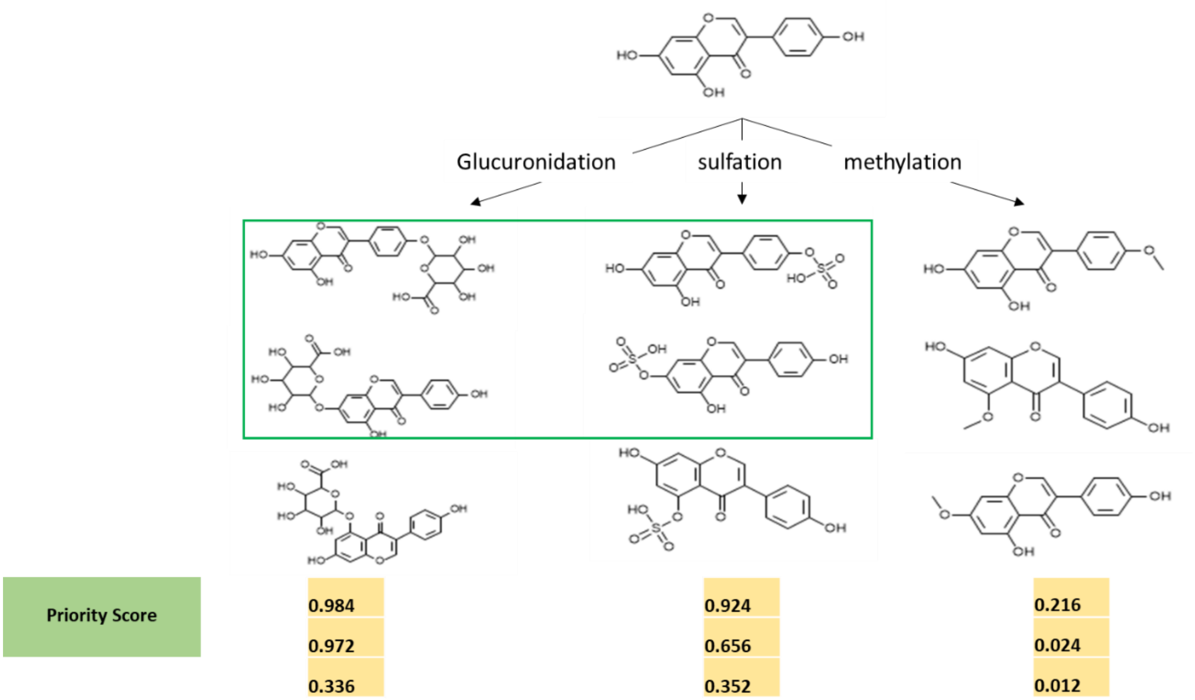

(B) Daidzein

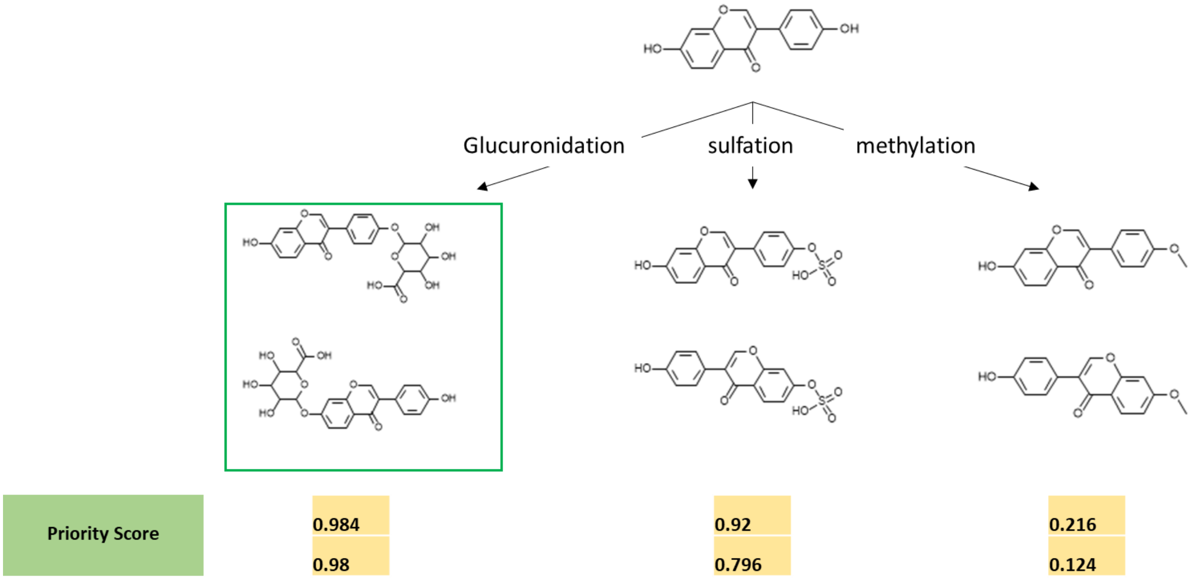

Supplementary Figure S2. Predicted metabolites of (A) genistein and (B) daidzein using Meteor Nexus 3.2.0, Nexus: 2.6.0. The prediction method options were: Site of Metabolism Scoring (with Molecular Mass Variance), with Molecular Mass Similarity Threshold of 70, a Scoring filter set at relative and a score threshold of 70.

(A) Genistein

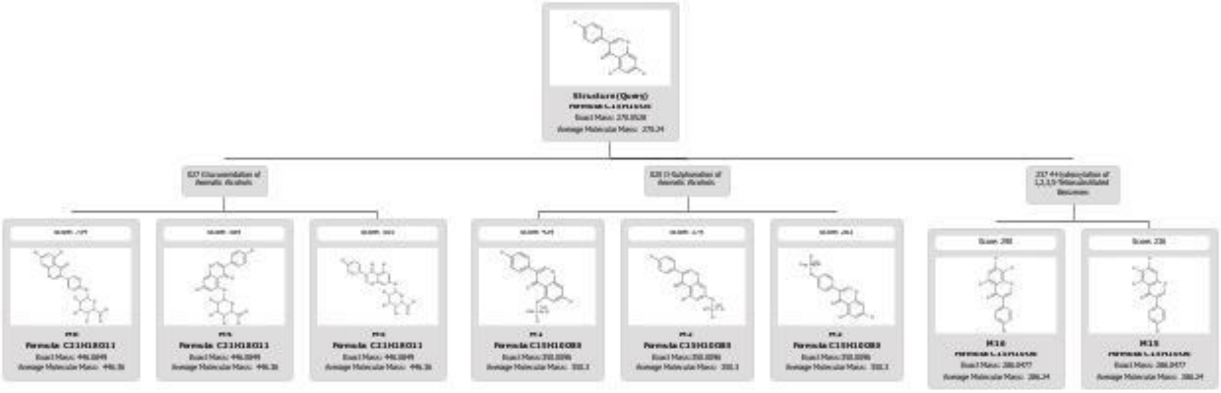

(B) Daidzein

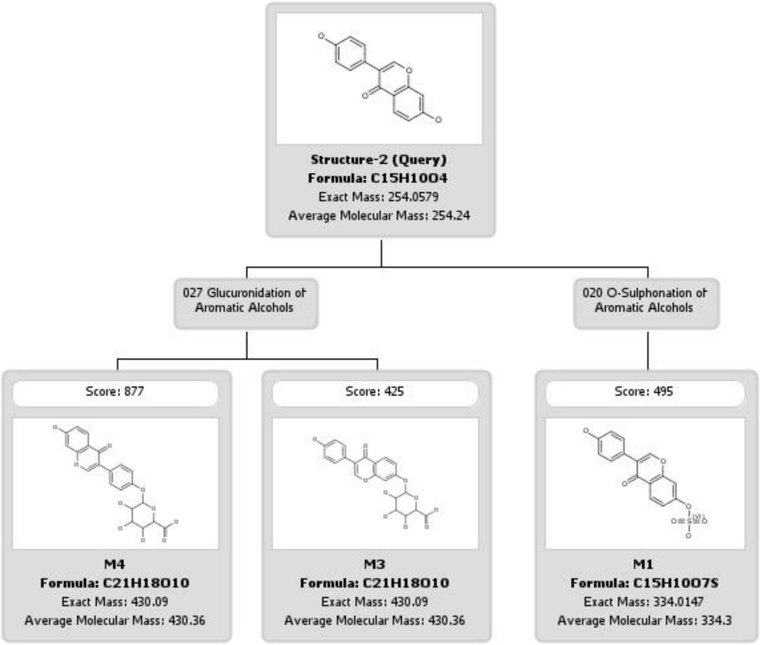

Supplementary Figure S3. Age and weight distribution of European population used for population PBPK modeling. Population PBPK modeling was applied to 100 individuals to consider individual anatomy and physiology variations. The age-based population was generated by scaling the age from 16 to 70, where the body weight ranged from 45 to 100 kg. The corresponding physiology and anatomy parameters were dependently ranged. This figure displays the range and counts of age and weight of human individuals.

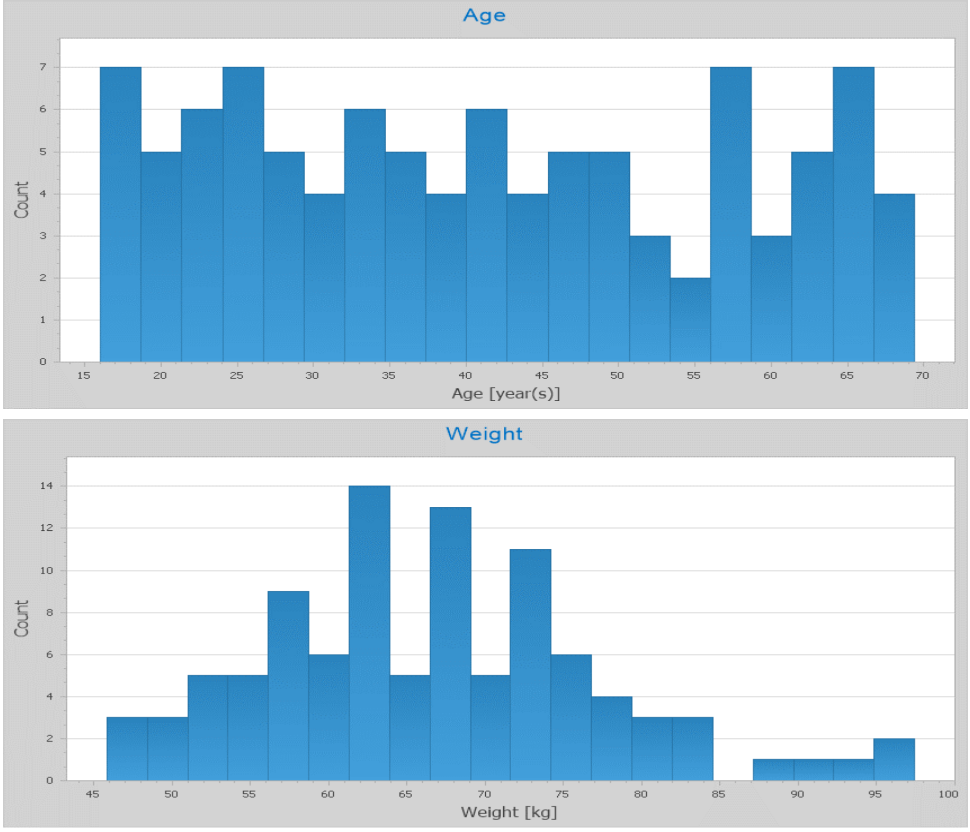

Supplementary Figure S4. Output of the uncertainty and sensitivity analyses of C_{max} for the rat oral PBPK models for (A) genistein and (B) daidzein. Sensitivity analysis results are presented as high (absolute value greater than or equal to 0.5), medium (absolute value greater than or equal to 0.2 but less than 0.5), or low (absolute value greater than or equal to 0.1 but less than 0.2); parameters with sensitivities less than 0.1 are not listed. Uncertainty analysis results are summarized as high uncertainty (value could be a factor of 2 or higher), medium uncertainty (value could be a factor between 0.3 and 2), or low uncertainty (value could be a factor of 0.3 or lower).

(A) Genistein

Genistein, Plasma Cmax		Uncertainty		
		High	Medium	Low
	High			Hepatic Clearance<br>pH (plasma)<br>Muscle Volume<br>Skin blood flow fraction<br>Dissolution time (50% dissolved)
	Medium			Muscle blood flow fraction<br>Dissolution shape
	Low			Hematocrit<br>Skin Volume<br>Lipophilicity<br>Lumen Stomach Gastric emptying

(B) Daidzein

Daidzein, Plasma Cmax		Uncertainty		
		High	Medium	Low
Sensitivity	High		Fraction unbound	Hepatic Clearance<br>pH (plasma)<br>Skin blood flow fraction<br>Lipophilicity<br>Dissolution time (50% dissolved)
	Medium			Muscle Volume<br>Muscle blood flow fraction
	Low			Dissolution shape<br>Skin Volume

Supplementary Figure S5. Simulated concentration-time profile of (A) genistein and (B) daidzein in rat plasma after repeated oral exposure of 0.3 mg/kg/day over 7 days.

(A)

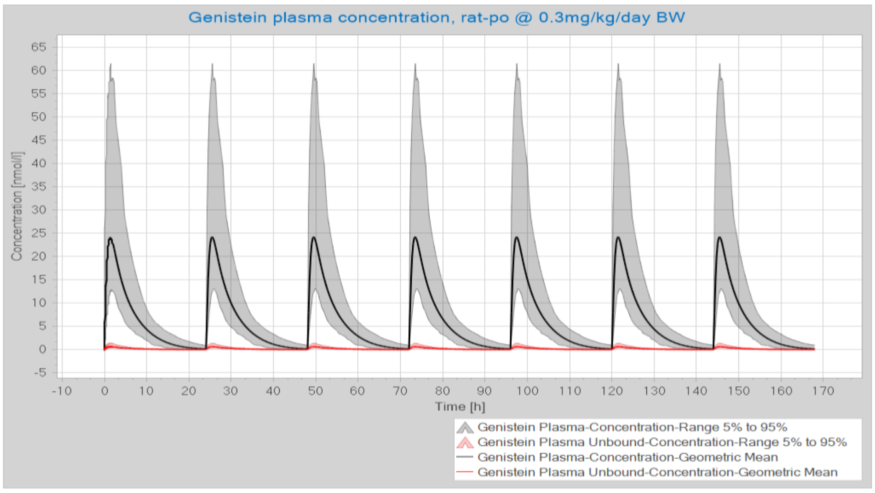

(B)

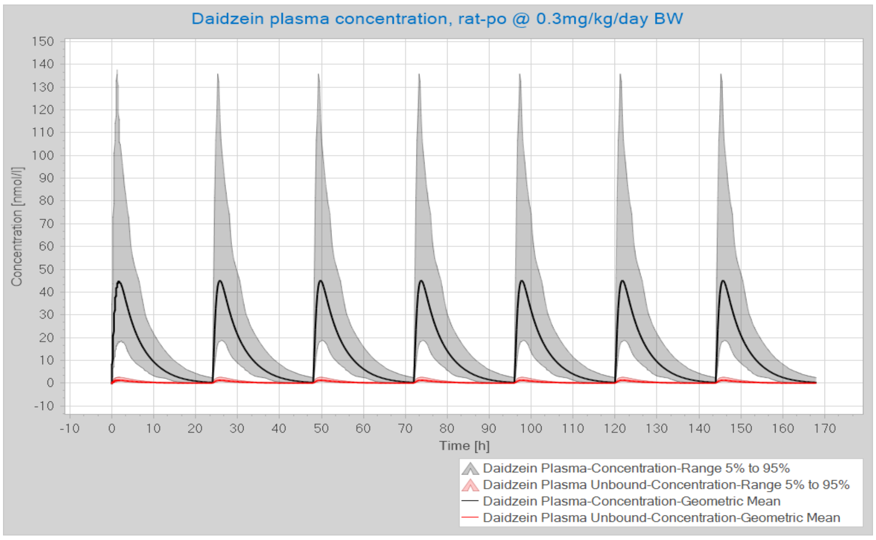

Supplementary Figure S6. Output of the uncertainty and sensitivity analyses of C_{max} for the human dermal PBPK models for genistein and daidzein. Sensitivity analysis results are presented as high (absolute value greater than or equal to 0.5), medium (absolute value greater than or equal to 0.2 but less than 0.5), or low (absolute value greater than or equal to 0.1 but less than 0.2); parameters with sensitivities less than 0.1 are not listed. Uncertainty analysis results are summarized as high uncertainty (value could be a factor of 2 or higher), medium uncertainty (value could be a factor between 0.3 and 2), or low uncertainty (value could be a factor of 0.3 or lower).

(A) Genistein

Genistein, Plasma Cmax		Uncertainty		
		High	Medium	Low
Sensitivity	High		Permeability across lipid bilayers (hydration-adjusted value)<br>Water solubility<br>Lipophilicity<br>First-pass skin_metabolism rate<br>SC Thickness	Skin blood flow fraction<br>Fraction unbound<br>Liver Volume<br>Skin temperature<br>Area of application<br>Mass dose per area
	Medium			Small Intestine Volume<br>Small Intestine Specific blood flow rate<br>pH (plasma)<br>Muscle blood flow fraction<br>Hematocrit<br>pKa value<br>Total Hepatic Clearance<br>protein/water partitioning coefficient<br>ED Thickness
	Low			Liver blood flow rate

(B) Daidzein

Daidzein, Plasma Cmax		Uncertainty		
		High	Medium	Low
Sensitivity	High	Permeability across lipid bilayers (hydration-adjusted value)	Skin temperature<br>SC Thickness	Skin blood flow fraction<br>Lipophilicity<br>Fraction unbound<br>Liver Volume<br>protein/water partitioning coefficient<br>Area of application<br>Mass dose per area
	Medium			Small Intestine Volume<br>Small Intestine Specific blood flow rate<br>pH (plasma)<br>Muscle blood flow fraction<br>Water solubility<br>pKa value<br>Total Hepatic Clearance<br>First-pass skin_metabolism rate
	Low			Liver blood flow rate<br>Large Intestine Volume<br>Large Intestine blood flow rate

References

Chen, Y., Jin, J. Y., Mukadam, S., Malhi, V., & Kenny, J. R. (2012). Application of IVIVE and PBPK modeling in prospective prediction of clinical pharmacokinetics: strategy and approach during the drug discovery phase with four case studies. *Biopharm Drug Dispos*, *33*(2), 85-98. https://doi.org/10.1002/bdd.1769

Davies, B., & Morris, T. (1993). Physiological parameters in laboratory animals and humans. *Pharm Res*, *10*(7), 1093-1095. https://doi.org/10.1023/a:1018943613122

de Bruyn Kops, C., Šícho, M., Mazzolari, A., & Kirchmair, J. (2021). GLORYx: Prediction of the Metabolites Resulting from Phase 1 and Phase 2 Biotransformations of Xenobiotics. *Chemical Research in Toxicology*, *34*(2), 286-299. https://doi.org/10.1021/acs.chemrestox.0c00224

Edginton, A. N., Schmitt, W., & Willmann, S. (2006). Development and evaluation of a generic physiologically based pharmacokinetic model for children. *Clin Pharmacokinet*, *45*(10), 1013-1034. https://doi.org/10.2165/00003088-200645100-00005

Jones, H., & Rowland-Yeo, K. (2013). Basic concepts in physiologically based pharmacokinetic modeling in drug discovery and development. *CPT Pharmacometrics Syst Pharmacol*, *2*(8), e63. https://doi.org/10.1038/psp.2013.41

Jones, H. M., Gardner, I. B., Collard, W. T., Stanley, P. J., Oxley, P., Hosea, N. A., Plowchalk, D., Gernhardt, S., Lin, J., Dickins, M., Rahavendran, S. R., Jones, B. C., Watson, K. J., Pertinez, H., Kumar, V., & Cole, S. (2011). Simulation of human intravenous and oral pharmacokinetics of 21 diverse compounds using physiologically based pharmacokinetic modelling. *Clin Pharmacokinet*, *50*(5), 331-347. https://doi.org/10.2165/11539680-000000000-00000

Kuepfer, L., Niederalt, C., Wendl, T., Schlender, J. F., Willmann, S., Lippert, J., Block, M., Eissing, T., & Teutonico, D. (2016). Applied Concepts in PBPK Modeling: How to Build a PBPK/PD Model. *CPT Pharmacometrics Syst Pharmacol*, *5*(10), 516-531. https://doi.org/10.1002/psp4.12134

Mordenti, J. (1986). Man versus beast: pharmacokinetic scaling in mammals. *J Pharm Sci*, *75*(11), 1028-1040. https://doi.org/10.1002/jps.2600751104

Nestorov, I. (2007). Whole-body physiologically based pharmacokinetic models. *Expert Opinion on Drug Metabolism & Toxicology*, *3*(2), 235-249. https://doi.org/10.1517/17425255.3.2.235

Sinha, V. K., Snoeys, J., Osselaer, N. V., Peer, A. V., Mackie, C., & Heald, D. (2012). From preclinical to human--prediction of oral absorption and drug-drug interaction potential using physiologically based pharmacokinetic (PBPK) modeling approach in an industrial setting: a workflow by using case example. *Biopharm Drug Dispos*, *33*(2), 111-121. https://doi.org/10.1002/bdd.1782

Valentin, J. (2002). Basic anatomical and physiological data for use in radiological protection: reference values. A report of age- and gender-related differences in the anatomical and physiological characteristics of reference individuals. ICRP Publication 89. *Ann ICRP*, *32*(3-4), 5-265.
